# Immune responses to *P falciparum* antibodies in symptomatic malaria patients with variant hemoglobin genotypes in Ghana

**DOI:** 10.1186/s12865-024-00607-1

**Published:** 2024-02-09

**Authors:** Kwame Kumi Asare, Benjamin Agrah, Fiifi Solomon Ofori-Acquah, William Kudzi, Nii Ayite Aryee, Linda Eva Amoah

**Affiliations:** 1 Department of Biomedical Science, School of Allied Health Sciences, University of Cape Coast, Cape Coast, Ghana; 2 Biomedical and Clinical Research Centre, College of Allied Health Sciences, University of Cape Coast, Cape Coast, Ghana; 3 Department of Medical Biochemistry, College of Health Sciences, University of Ghana Medical School, University of Ghana, Korle- Bu, Accra, Ghana; 4 West Africa Genetic Medicine Centre, University of Ghana, Accra, Ghana; 5 Department of Immunology, Noguchi Memorial Institute for Medical Research, University of Ghana, Accra, Ghana

**Keywords:** Heamoglobin S and C, Salivary gland antigen, Immunoglobulin G, Symptomatic malaria, Capillarys 2 flex piercing analyzer

## Abstract

**Background:**

Haemoglobin (Hb) variants such as sickle cell trait (SCT/HbAS) play a role in protecting against clinical malaria, but little is known about the development of immune responses against malaria parasite (*Plasmodium falciparum* surface protein 230 (Pfs230) and *Plasmodium falciparum* erythrocyte binding antigen 175 region-3 (PfEBA175-3R)) and vector (on the *An. gambiae* Salivary Gland Protein-6 peptide 1 (gSG6-P1)) antigens in individuals with variants Hb genotypes. This study assessed antibody (IgG) responses against malaria parasite, *Pfs230* and PfEBA175-3R and vector, *gSG6-P1* in febrile individuals with variant Hb genotypes.

**Methods:**

The study was conducted on symptomatic malaria patients attending various healthcare facilities throughout Ghana. Microscopy and ELISA were used to determine the natural IgG antibody levels of gSG6-P1, PfEBA175-3R & Pfs230, and Capillarys 2 Flex Piercing was used for Hb variants determination.

**Results:**

Of the 600 symptomatic malaria patients, 50.0% of the participants had malaria parasites by microscopy. The majority 79.0% (398/504) of the participants had Hb AA, followed by HbAS variant at 11.3% (57/504) and HbAC 6.7% (34/504). There were significantly (*p* < 0.0001) reduced levels of gSG6-P1 IgG in individuals with both HbAC and HbAS genotypes compared to the HbAA genotype. The levels of gSG6-P1 IgG were significantly (*p* < 0.0001) higher in HbAS compared to HbAC. Similarly, Pfs230 IgG and PfEBA-175-3R IgG distributions observed across the haemoglobin variants were significantly higher in HbAC relative to HbAS.

**Conclusion:**

The study has shown that haemoglobin variants significantly influence the pattern of anti-gSG6-P1, Pfs230, and PfEBA-175 IgG levels in malaria-endemic population. The HbAS genotype is suggested to confer protection against malaria infection. Reduced exposure to infection ultimately reduces the induction of antibodies targeted against *P. falciparum* antigens.

## Background

Malaria continues to be a significant public health problem in Ghana, as it is in the rest of Sub-Saharan Africa. Ghana is one of ten African countries with the highest malaria burden with 5.9 million, 5.7 million, and 5.3 million malaria cases and 39,214, 12,557 and 11,557 malaria related death in 2020, 2021 and 2022 respectively [1–3]. Malaria is an endemic disease in Ghana, with seasonal changes in the north. The southern and middle belts have two different rainy seasons, but the northern region has a single rainy season that lasts from May to September. The transmission time in the northern section is six to seven months, and in the higher part, it is three to four months. Between July and November, the most malaria cases occur. Children under the age of five, as well as pregnant women, are more vulnerable due to weakened immunity. Malaria transmission in the southern belt lasts nine months or more.

Global efforts to control malaria shifted from reducing malaria morbidity and mortality to targeting the eradication of malaria [4, 5]. And in pursuit of this objective, several vaccine research efforts have focused on the pre-erythrocytic stage or transmission-blocking and blood-stage vaccine development but have encountered setbacks due to the redundancy in the invasion pathways and polymorphic nature of parasites’ antigens [6, 7]. Although haemoglobin variants such as sickle cell trait (SCT) are known to play a role by conferring protection against clinical malaria, little or no information about the effects of haemoglobin variants have on the production of malaria parasite antibodies [8, 9].

Malaria has had a considerable effect on human population genetics and continues to do so since traits conferring partial tolerance to infection or disease progression are selected in endemic areas [10]. This malaria hypothesis has been validated for several genetic polymorphisms such as HbS/C genotypes, and it is well established that hemoglobin variants, specifically HbAS, can confer relative malaria resistance [11–14].

HbAS has been associated with a high proportion of polyclonal *P. falciprum* infections, suggesting an increased breadth of antibody responses [9, 15]. It has been proposed that the protective effect of HbAS’s is related to multiple complex mechanisms linked with the immune response to malaria [9]. Populations living in malaria endemic have a variety of hemoglobin variants that may work synergistically to reduce *Plasmodium* virulence in humans and result from various geographical founding effects. Consequently, the diagnosis of parasites in febrile malaria patients provides knowledge that assists malaria-endemic countries in assessing their place on the spectrum of malaria elimination [3].

Moreover, antibodies against various *P. falciprum* antigens are crucial for controlling and managing parasite burden and disease progression. The prevalence of IgG of gSG6-P1, PfEBA175, and Pfs230 varies among individuals based on exposures and the level of malaria transmission among communities [16–18]. Haemoglobin (Hb) interacts with the innate immune system directly or through binding to pathogen-associated molecular patterns (PAMPs) [19]. In the search for immunological surrogates of immunity against malaria, a plethora of research has focused on antibody levels without determining the Hb genotype of the individuals, which is undoubtedly a critical parameter in developing immunity to malaria.

There is, thus, the need to know whether variant haemoglobin genotypes affect the development of antibody responses against malaria antigens. Although some antibodies have been identified as key to malaria protection and alleviating symptoms of the disease, there is a lack of rigorous information on the influence of haemoglobin variants on IgG levels in symptomatic patients across Ghana. The study determined the levels of antimalarial antibodies in symptomatic malaria patients with variant Hb genotypes (HbAS and HbAC).

## Methods

### Study design and study population

The study was carried out among symptomatic malaria patients seeking treatment at randomly selected healthcare facilities in the ten regions of Ghana. The enrolled suspected malaria patients (*n* = 600) were between the ages of 1 to 88 years. The samples were conveniently selected from 300 samples that tested positive and 300 samples that tested negative for *P. falciparum* by microscopy. Approximately, 1 ml of venous blood was collected from each participant into EDTA vacutainer tubes for malaria and mosquito antibody level estimation and haemoglobin phenotyping.

### Microscopy

Thin and thick blood smears were prepared using 2 µL and 6 µL respectively of whole blood and processed as previously described [20]. Two independently WHO-certified microscopists examined the slides using an x100 oil immersion microscope.

### Indirect enzyme-linked immunosorbent assay (ELISA)

An indirect ELISA was used to quantify the Immunoglobin G (IgG) antibodies levels against gSG6-P1, Pfs 230 and EBA-175 antigens among the participants using a previously published protocol [21, 22]. A 96-well NUNG Maxisorp ELISA plate was coated with either 1 µg/well of gSG6-P1 in phosphate buffer saline (PBS, pH 7.2), 20 µl/well EBA-175 in PBS, pH 7.2 or 1 µg/well of Pfs230 in carbonate buffer, pH 9.0 and incubated overnight at 4°C. The samples used include diluted plasma (1:200), a positive control sample obtained from a pool of seropositive individuals and negative control samples obtained from various pools of seronegative individuals in duplicate. The plates were washed three times and incubated for 1 hour with 100 µl/well of 1:3000 dilution of goat antihuman IgG-HRP. The plates were incubated with peroxidase substrate 3,3’,5,5’-teramethylbenzidine (TMB) for 10 min. 100 µl of 0.2 mM sulfuric acid was added to halt the enzymatic reactions and the optical densities (OD) of the contents in the wells were read at 450 nm using a Multiskan FC plate reader (Thermo Scientific, USA).

### Haemoglobin genotyping

Two methods were used for haemoglobin genotyping. Part of the samples were analyzed using the automated multi-assay analyzer, the Capillarys 2 Flex Piercing ® [23, 24]. The analyzer was calibrated using a Hb A1c package (Sebia, Lisses, France. The samples were vortexed and mixed for 5 s and applied in a conical tube and barcoded for easy identification before sliding the sample rack for analysis. All sample preparations and analyses were performed in compliance with the manufacturer’s instructions. The second method used for Hb genotyping was based on the most commonly used method, the cellulose acetate electrophoresis at alkaline pH using standard procedure. Known samples of HbA, HbS, HbC and HbF were used as controls for each gel that was ran.

### Data analysis

The information was entered into Excel and analyzed with GraphPad Prism (v.9.5.0). The data were grouped according to regions, diagnostic tests, haemoglobin genotypes, gender and age categories. The infection frequency was represented by simple counts and proportions. The IgG antibodies were categorized into 0-999, 1000–1999, 2000–2999 and > 3000 ng/mL for gSG6-P1 and Pfs230 IgG antibodies and 0-1999, 2000–4999, 5000–9999 and > 10,000 ng/mL for PfEBA175 3R IgG antibodies and analyzed using median and ranges [24]. The pattern of antibody distribution between *P. falciprum* positive and negative infections by microscopy were assessed using the odds ratio (OR). The statistical significance in the antibody distribution across haemoglobin (Hb) phenotypes was analyzed using the Chi-square test. Wilcoxon signed-rank test was used to assess the statistical significance in the distribution of antibodies across Hb genotypes. Statistical significance was set at *P* < 0.05.

## Results

### Characteristics of the study participants

the mean age ± SEM for the 600 study participants was 21.84 ± 0.80 years. A total of 57.3% of the population were female. *Plasmodium falciparum* was identified in 300 participants (50.0%). The highest (229,452, SE = 72,305) and lowest (35,217, SE = 11,091) mean parasite densities were obtained in study participants from the Upper West region and Central region respectively. Hb genotyping data were available for 504 (84%) of the population. The majority 398/504 (79.0%) of the participants had normal haemoglobin genotype (HbAA), followed by HbAS variant at 57/504 (11.3%) and HbAC 34/504 (6.7% ) (Table 1). Due to the low prevalence of the variant haemoglobin genotypes HbAS and HbAC, the data was analyzed at the national level rather than regional.

**Table 1:** Demographic characteristics of the study subjects according to regions of Ghana

	Region of Ghana										
Demographic characteristic	AR	BAR	CR	ER	GAR	NR	UER	UWR	VR	WR	Total
Age, years, mean ± SEM (95% CI)	20 ± 2.459 (15.65–25.49)	19.40 ± 2.39 (14.62–24.18)	23.98 ± 2.558 (18.87–29.10)	19.75 ± 2.411 (14.93–24.57)	27.75 ± 2.167 (23.41–32.09)	22.85 ± 3.053 (16.74–28.96)	22.90 ± 2.424 (18.05–27.75)	21.15 ± 2.595 (15.96–26.34)	21.80 ± 3.08 (15.64–27.96)	18.27 ± 1.862 (14.54–21.99)	21.84 ± 0.799 (20.27–23.41)
Sex (Female/Male), n	60 (35/25)	60 (27/33)	60 (33/27)	60 (37/23)	60 (34/26)	60 (31/29)	60 (40/20)	60 (36/24)	60 (35/25)	60 (36/24)	600 (344/266)
Diagnosis, n/N (%)											
Microscopy +	30/60 (50.0)	30/60 (50.0)	30/60 (50.0)	30/60 (50.0)	30/60 (50.0)	30/60 (50.0)	30/60 (50.0)	30/60 (50.0)	30/60 (50.0)	30/60 (50.0)	300/600 (50.0)
Microscopy -	30/60 (50.0)	30/60 (50.0)	30/60 (50.0)	30/60 (50.0)	30/60 (50.0)	30/60 (50.0)	30/60 (50.0)	30/60 (50.0)	30/60 (50.0)	30/60 (50.0)	300/600 (50.0)
Parasite density/uL Mean (SEM)	55,346 (13,423)	85,122 (30,345)	35,217 (11,091)	181,296 (44,939)	35,911 (9309)	178,747 (34,438)	104,809 (20,736)	229,452 (72,305)	48,254 (10,932)	47,582 (8277)	104,392 (11,508)
Antibodies, median (Range), ng/mL											
** gSG6-P1**											
	1234.0(0.0-4842.0)	1196.0(455.6-35730.0)	1394.0 (0.0-4739)	1297.0 (0.0-3364.0)	1265 (0.0-5866.0)	1117.0 (459.2–3979.0)	1125.0 (415.4–2340.0)	1953.0 (0.0-5114.0)	1001.0 (395.2–5697.0)	1049.0 (627.5–4936)	1352.0(0.0-35730.0)
**Pfs230**											
	4799.0 (0.0-31670.0)	1891.0 (550.7–7429.0)	1932.0 (664.2–7236.0)	1550.0 (0.0-12252.0)	1392.0 (0.0-4538.0)	2506.0 (0.0-34074.0)	2392.0 (0.0-8597.0)	2392.0 (964.4-14055.0)	2267.0 (927.5-15022.0)	2630.0 (0.0-15504.0	2140.0 (0.0-34074.0)
**PfEBA175-3R**											
	7555.0 (0.0-106964.0)	6535.0 (1899.0-77800.0)	5699.0 (860.7-53898.0)	5618.0 (860.7-216558.0)	4276.0 (0.0-38402.0)	19423.0 (0.0-8433172.0)	4712.0 (909.8-38258.0)	10417.0 (0.0-241151.0	8863.0 (0.0-335362.0)	5000.0 (0.0-247994.0)	6769.0 (0.0-8433172.0)
Hb Genotypes, n/N (%)											
AA	44/52 (84.6)	41/52(78.8)	40/53 (75.5)	51/60 (85.0)	38/43 (88.4)	44/55 (80.0)	18/26 (69.2)	43/51 (84.3)	46/54 (85.1)	33/58 (56.9)	398/504 (79.0)
AC	2/52 (3.8)	6/52 (11.5)	4/53 (7.5)	2/60 (3.3)	1/43 (2.3)	5/55 (9.1)	4/26 (15.4)	5/51 (9.8)	3/54 (5.6)	2/58 (3.4)	34/504 (6.7)
AF	1/52 (1.9)	-	1/53 (1.9)	1/60 (1.7)	-	-	-	-	-	1/58 (1.7)	4/504 (0.8)
AS	5/52 (9.6)	3/52 (5.8)	7/53 (13.2)	6/60 (10.0)	4/43 (9.3)	4/55 (7.3)	1/26 (3.8)	2/51 (3.9)	4/54 (7.4)	21/58 (36.2)	57/504 (11.3)
CC	-	-	-	-	-	2/55 (3.6)	-	1/51 (2.0)	-	-	3/504 (0.6)
CS	-	2/52 (3.8)	1/53 (1.9)	-	-	-	-	-	-	-	3/504 (0.6)
SS	-	-	-	-	-	-	-	-	1/54 (1.9)	-	1/504 (0.2)
ACF	-	-	-	-	-	-	3/26 (11.5)	-	1/54 (1.9)	-	4/504 (0.8)

*CI* Confidence interval, *SEM* Standard error of the mean, n positive samples, *N* total samples tested, *AR* Ashanti region, *BAR* Brong Ahafo region, *CR* Central region, *ER* Eastern region, *GAR* Greater Accra region, *NR* Northern region, *UER* Upper East regon, *UWR* Upper Weat region, *VR* Volta region, *WR* Western region

### Parasite density and haemoglobin genotypes

**Fig. 1:**
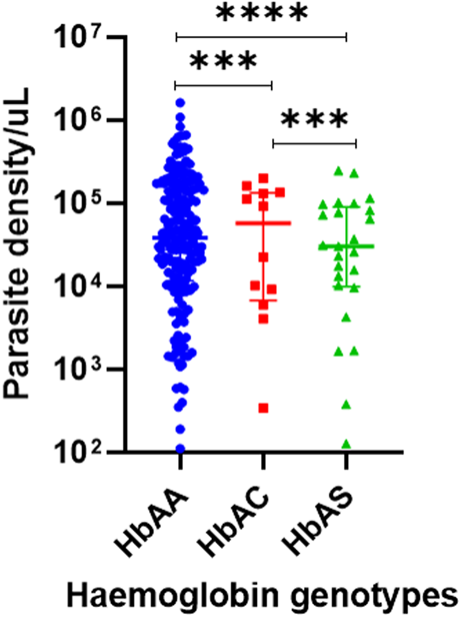
Distribution of parasite density among haemoglobin variants

Malaria parasite density was significantly higher in samples with haemoglobin AA compared to haemoglobin AC, *p* = 0.001 and AS, *p* < 0.001. similarly, malaria parasite density in samples with haemoglobin AS was significantly (*p* = 0.001) lower than the densities in haemoglobin AC samples (Fig. 1).

### *Anopheles* and *Plasmodium falciparum* antibody levels across the study population

The seroprevalence of gSG6-P1 was 223/399 (55.9% ), 34/57 (59.6% ) and 17/35 (48.6%) among microscopy-positive individuals with HbAA, HbAS and HbAC respectively. Similar seroprevalence patterns were observed for Pfs230 IgG and PfEBA-175-3R IgG antibodies in individuals with HbAA, HbAS and HbAC. The odds of detecting gSG6-P1 IgG antibodies among individuals that tested positive and negative by microscopy was 0.944 (0.431–2.052) and 1.478 (0.783–1.278) in Hb AC and Hb AS respectively. Similar observations were made for Pfs230 IgG and PfEBA-175-3R IgG antibodies in individuals with variant haemoglobin genotypes (Table 2).

**Table 2:** Pattern of *P. falciprum* malaria diagnosis by microscopy across vector and parasites antibody levels

	Prevalence n/N (%)		
	Microscopy+	Microscopy-	OR (95% CI)
**gSG6-P1 IgG**			
HbAA	223/399 (55.9)	169/399 (42.4)	1.32 (1.033 to 1.679)
HbAC	17/35 (48.6)	18/35 (51.4)	0.944 (0.431 to 2.052)
HbAS	34/57 (59.6)	23/57 (40.4)	1.478 (0.783 to 1.278)
**Pfs230 IgG**			
HbAA	222/399 (55.6)	165/399 (41.4)	1.345 (1.05 to 1.717)
HbAC	17/35 (48.6)	18/35 (51.4)	0.944 (0.431 to 2.052)
HbAS	33/57 (57.9)	22/57 (38.6)	1.50 (0.782 to 2.822)
**PfEBA-175-3R IgG**			
HbAA	222/399 (55.6)	165/399 (41.4)	1.345 (1.05 to 1.717)
HbAC	16/35 (45.7)	18/35 (51.4)	0.889 (0.397 to 1.957)
HbAS	33/57 (57.9)	23/57 (40.4)	1.435 (0.755 to 2.66)

### Age and sex stratified *Anopheles* and *Plasmodium falciparum* IgG concentrations

**Fig. 2:**
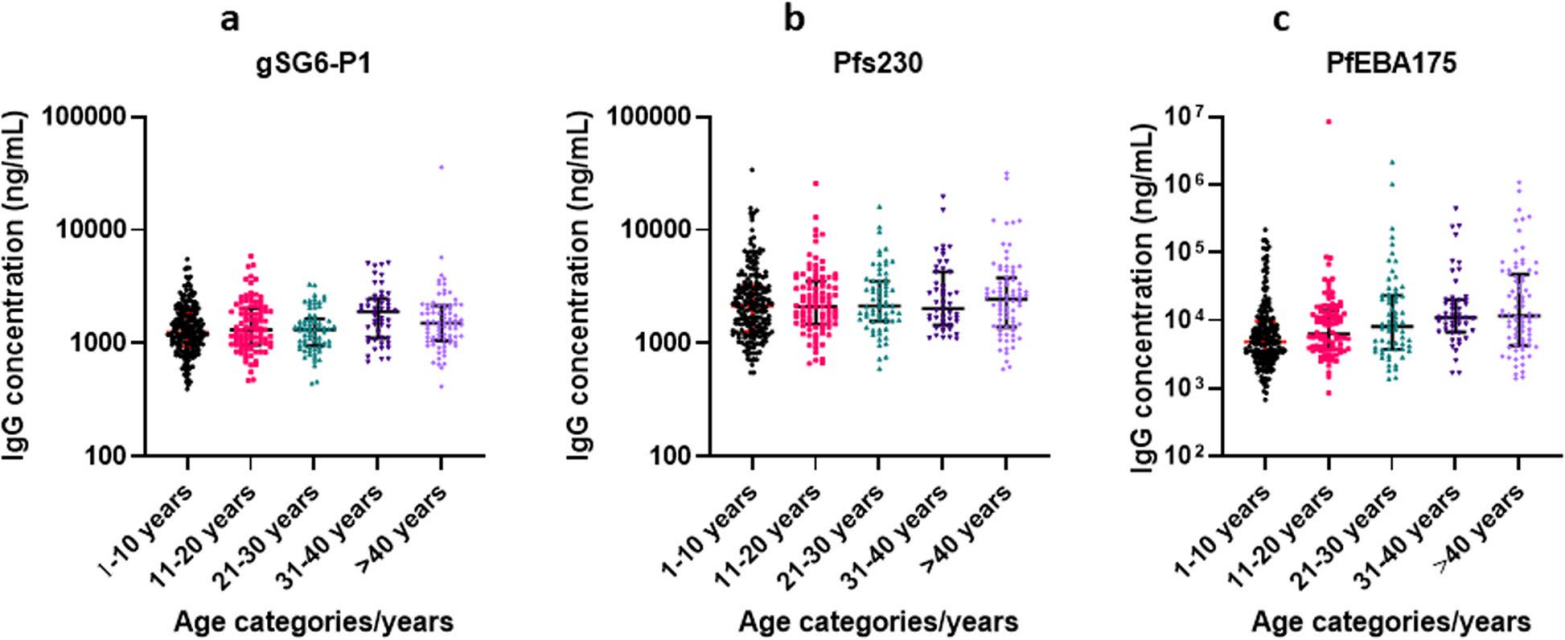
IgG antibody distribution across age categories. **a** gSG6-P1 IgG; **b** Pfs230 IgG; **c** PfEBA-175 IgG

**Fig. 3:**
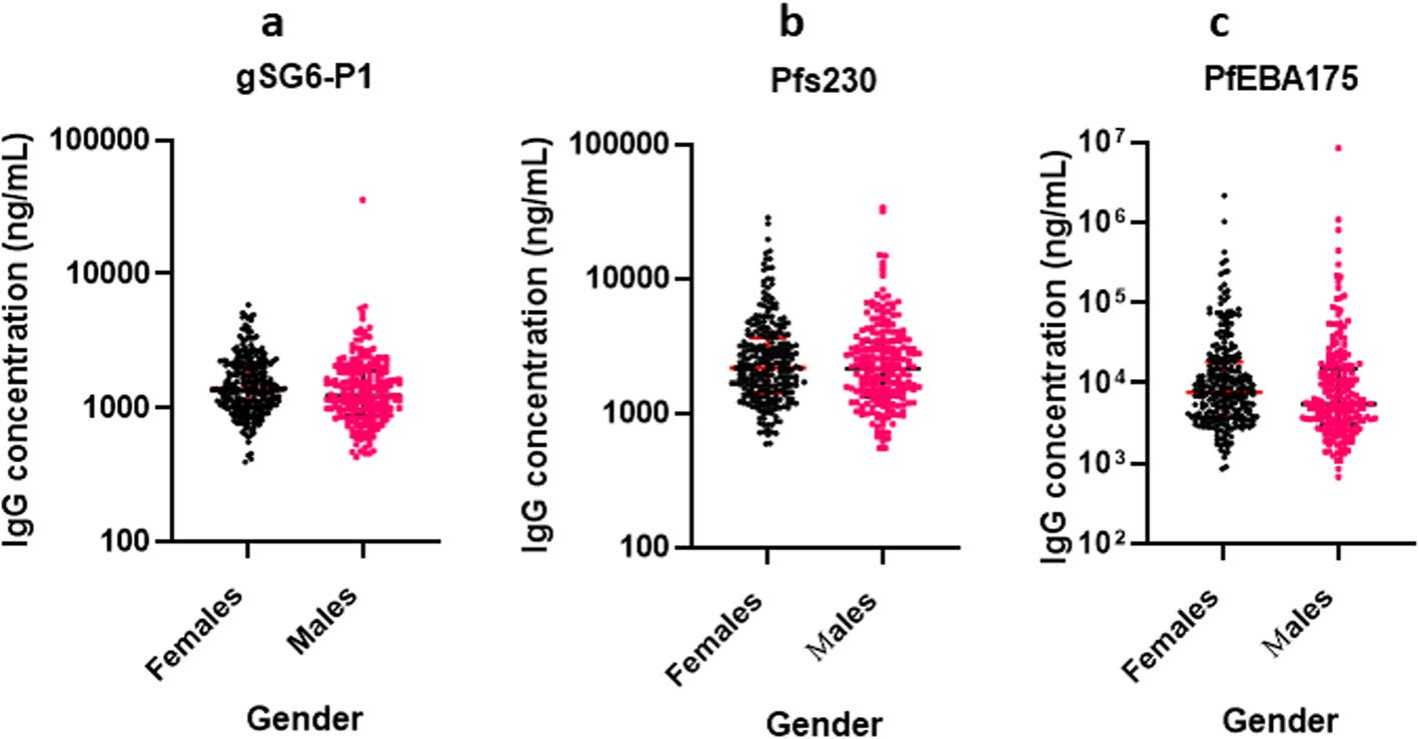
IgG antibody distribution across gender. **a** gSG6-P1 IgG among males and females; **b** Pfs230 IgG among males and females; **c** PfEBA-175 IgG among males and females

The median (95% CI) concentration of the gGS6-P1 IgG among the age 1–10, 11–20, 21–30, 31–40 and > 40 years were 1,245 (1,159-1,384), 1,306 (1,131-1,565), 1,314 (1,105-1,415),1,891 (1,720-2,411) and 1,490 (1,211-2,952) ng/mL respectively. There was a significant difference in the level of gSG6-P1 IgG antibodies between 1 and 10 years & 31–40 years, *p* < 0.001; 11–20 years & 31–40 years, *p* < 0.001; and 21–30 years & 31–40 years, *p* < 0.001 (Fig. 2a). There were no significant differences between age categories and the distribution of Pfs230 IgG except that responses in 1–10 years were less than in the > 40 years, *p* = 0.012 (Fig. 2b). Also, there were significant differences in the distribution of PfEBA-175 3R IgG between 1 and 10 years & 31–40 years, *p* = 0.011; 1–10 years & > 40 years, *p* = 0.001; 11–20 years & 21–30 years, *p* = 0.033; 11–20 years & 31–40 years, *p* = 0.014 and 11–20 years & > 40 years, *p* < 0.001 (Fig. 2c). There was a significant difference in levels of gSG6-P1 IgG antibodies between females and males, *p* = 0.012 (Fig. 3a). Pfs230 IgG (*p* = 0.846) levels showed no significant difference between female and male participants (Fig. 3b) and similarly, there was no significant difference in the PfEBA-175 3R IgG (*p* = 0.367) levels among female and male participants (Fig. 3c).

### gSG6-P1, Pfs230 and PfEBA-175-3R IgG concentrations and haemoglobin genotype

**Fig. 4:**
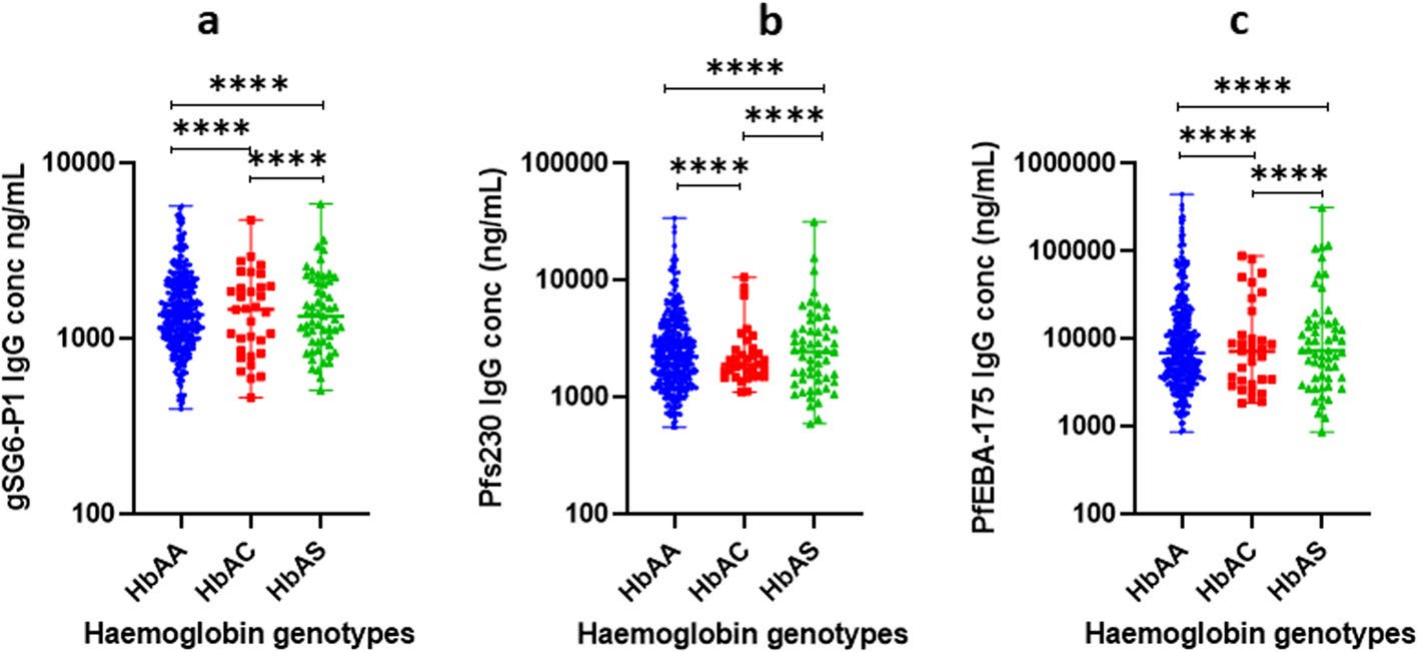
Distribution of gSG6-P1 IgG antibody concentrations across haemoglobin variants. **a** The gSG6-P1 IgG distribution among Hb AA, Hb AC, Hb AS genotypes. **b** The Pfs230 IgG distribution among Hb AA, Hb AC, Hb AS genotypes. **c** The PfEBA-175-3R IgG distribution among Hb AA, Hb AC, Hb AS genotypes The data is presented as median and 95% CI; Statistical analysis was performed using the Wilcoxon rank test, ns, *p* >0.05; *, *p *< 0.05; **, *p* < 0.01; ***, *p* < 0.001; ****, *p* < 0.0001

The results showed significantly reduced levels of gSG6-P1 IgG in HbAC, and HbAS, (*p* < 0.001) genotypes compared to the HbAA genotype. Also, the levels of gSG6-P1 IgG were significantly reduced in HbAC compared to HbAS, *p* < 0.001 (Fig. 4a). There were significantly reduced levels of Pfs230 IgG IgG in HbAC, and HbAS, (*p* < 0.001) genotypes compared to the HbAA genotype. Also, the levels of Pfs230 IgG IgG were significantly reduced in HbAC compared to HbAS, *p* < 0.001 (Fig. 4b). There were significantly reduced levels of PfEBA-175-3R IgG in HbAC, and HbAS, (*p* < 0.001) genotypes compared to the HbAA genotype. Also, the levels of PfEBA-175-3R IgG were significantly reduced in HbAC compared to HbAS, *p* < 0.001 (Fig. 4c).

### Haemoglobin variants and the pattern of *P. falciparum* IgG antibodies in individuals that tested positive for *P. falciparum* by microscopy

The proportion of the haemoglobin genotypes between *P. falciprum* positive infection by microscopy and *P. falciprum* negative by microscopy showed the distribution of Hb genotype were the same. The difference in the proportion between microscopy positve (+) and microscopy negative (-) were 0.47% in HbAA, 2.08% in HbAC and 6.99% in HbAS. There were no significant differences in the prevalence Hb genotypes between microscopy positive and negative individuals (Table 3). Comparing the proportion of the *P. falciprum* infection in Hb AA and other Hb genotypes showed a highly significant difference in the proportions of *P. falciprum* infection in other Hb genotypes compared to Hb AA. The difference in the proportion of *P. falciprum* infections between HbAA and HbAC is 73.1% (×2 = 316.69, *p* < 0.001) and HbAA and HbAS is 67.24% (×2 = 264.07, *p* < 0.0001) (Table 4). The mean *P. falciprum* IgG antibodies were lower in HbAC (Pfs230 IgG = 2149 and PfEBA175-3R = 6551) compared to HbAA (Pfs230 = 3073 and PfEBA175-3R = 10,651). The HbAS on the other hand had a higher mean *P. falciprum* IgG antibodies (Pfs230 = 3530 and PfEBA175-3R = 11,470) compared to HbAA (Table 5).

**Table 3:** Comparison of proportion of Hb genotypes between *P. falciprum* positive and negative infection by Microscopy

Hb Genotypes	Microscopy +	Microscopy -	Diff % (95% CI)	Chi-square	*p*
HbAA	229/290 (78.96%)	170/214 (79.43%)	0.47% (-6.84–7.49%)	0.016	0.897
HbAC	17/290 (5.86%)	17/214 (7.94%)	2.08% (-2.35–6.99%)	0.845	0.358
HbAS	34/290 (11.72%)	23/214 (10.75%)	0.97% (-4.86–6.43%)	0.115	0.734
HbACF	3/290 (1.03%)		1.03% (-0.86–2.99%)	2.213	0.137
HbAF	3/290 (1.03%)	1/214 (0.47%)	0.56% (-1.68–2.55%)	0.49	0.484
HbCC	1/290 (0.34%)	2/214 (0.93%)	0.59% (-1.13–3.01%)	0.729	0.393
HbSC	2/290 (0.69%)	1/214 (0.47%)	0.22% (-1.97–2.05%)	0. 1	0.752
HbSS	1/290 (0.34%)		0.34% (-1.45–1.92%)	0.728	0.394

**Table 4:** Comparison of proportion of *P. falciprum* infection in HbAA and other Hb genotypes

Hb Genotypes	Microscopy +	Diff % (95% CI)	Chi-square	*p*
HbAA	229/290 (78.96%)	Ref		
HbAC	17/290 (5.86%)	73.10% (67.05 to 77.91%)	316.69	< 0.0001
HbAS	34/290 (11.72%)	67.24% (60.66–72.60%)	264.07	< 0.0001
HbACF	3/290 (1.03%)	77.93% (72.51–82.28%)	366.29	< 0.0001
HbAF	3/290 (1.03%)	77.93% (72.51–82.28%)	366.29	< 0.0001
HbCC	1/290 (0.34%)	78.62% (73.32–82.92%)	373.91	< 0.0001
HbSC	2/290 (0.69%)	78.27% (72.91–82.59%)	370.03	< 0.0001
HbSS	1/290 (0.34%)	78.62% (73.32–82.92%)	373.91	< 0.0001

**Table 5:** The mean concentration of *P. falciprum* IgG antibodies across Hb genotypes

		IgG antibody concentration, mean, S.E (95% CI)		
Hb genotypes	Microscopy +	gSG6-P1	Pfs230	PfEBA175-3R
HbAA	229/290 (78.96%)	1507, 61.92(1385–1629)	3073, 255.5 (2570–3577)	10,651, 957.9 (876-12539)
HbAC	17/290 (5.86%)	1608, 192.4 (1200–2015)	2149, 443.3 (1210–3089)	6551, 1827 (2678–10,423)
HbAS	34/290 (11.72)	1494, 142.0 (1205–1783)	3530, 912.3 (1674–5386)	11,470, 3343 (4668–18,273)
HbACF	3/290 (1.03)	934.7, 218.6 (-5.743-1875)	3691, 1498 (-2756-10138)	4680, 596.6 (2113–7247)
HbAF	3/290 (1.03)	936.4, 626.7 (-1760-3633)	5481, 4320 (-13108-24069)	8626, 2390 (-1656-18908)
HbCC/HbSC/HbSS	4/290 (1.37)	2071, 683.6 (-104.5-4246)	2753, 350.1 (1639–3867)	16,348, 9756 (-14699-47395)

## Discussion

The study assessed the haemoglobin variants (sickle cell trait and haemoglobin C trait) on the *An. gambiae* Salivary Gland Protein-6 peptide 1 (gSG6-P1), *Plasmodium falciparum* surface protein 230 (Pfs230) and *Plasmodium falciparum* erythrocyte binding antigen 175 region-3 (PfEBA175-3R) immune responses among symptomatic malaria infection in Ghana.

The identification of biomarkers for assessing potential exposure to vector bites and the risk of vector-borne infections have recently gained substantial importance [25, 26]. Previous reports showed that *Plasmodium falciparum-*infected individuals presented with higher levels of IgG response against gSG6-P1 antigen than the uninfected individuals [25, 27–29]. Generally, the average time for outdoor activities has decreased over the years across ages due to work, rest, and leisure pursuits impacted by technological advancement [30]. The outdoor time among age groups under 30 years has decreased by 20% compared to age group 31 to 50 years decreased by only 9% [31, 32]. Thus, the age group 31–40 years age group likely spent more time in outdoor activities that exposed them to mosquito bites and increased the level of IgG concentration of gSG6-P1 among them compared to those under 30 years. The study observed high seroprevalence of IgG response against gSG6-P1 antigen in HbAA and HbAS participants among *P. falciprum* positive infections by microscopy compared to participants with submicroscopic positive *P. falciprum* infections. Contrarily, the participants with HbAC had a high seroprevalence of IgG response against gSG6-P1 antigen in the samples with *P. falciparum* negative infection by microscopy compared to *Plasmodium falciparum* positive infection samples diagnosed with microscopy.

The study showed that the HbAC and HbAS have a reduced distribution of gSG6-p1 IgG antibodies compared to HbAA. The observation could partly explain the lower IgG gSG6-P1 antibodies having a significant association with *P. falciprum* infection by microscopic diagnosis. High IgG antibody levels are associated with malaria infection, whereas Hb traits such as HbAS and Hb AC protect against *P. falciprum* infection [33]. Thus, the low IgG levels in the Hb traits explain the counter effect haemoglobin variants have on antibody development. The gSG6-p1 IgG-specific antibodies were detected, and no screening was performed to test for cross-reactivity with other antigens from other pathogens. Currently, no study has reported on the cross-reactivity of gSG6-p1 IgG-specific antibodies to non-mosquito antigens [34, 35].

A significantly lower median of anti-Pfs230-specific IgG were observed in HbAC and Hb AS compared to HbAA. HbAS showed a significantly high IgG response to Pfs230 antigens when compared with HbAC. Both haemoglobin variants C and S are shown to protect against severe malaria [36]. Hb AC is associated with frequent patent carriage of parasites and gametocytes [36, 37]. Two independent studies from Ghana and Burkina Faso reported higher gametocyte carriage in HbAC when compared with HbAA individuals [38, 39]. The Pfs230 antigen is expressed on the surface of gametocytes and only becomes exposed to the host immune system upon destruction and rapture from erythrocytes. The low anti-Pfs230 IgG indicates reduced destruction of gametocytes in HbAC variants. Thus, higher gametocyte carriage in HbAC may be due to reduced gametocyte destruction and relatively produced patent gametocytes [38, 40]. The haemoglobin C is associated with a longer *P. falciprum* infection due to a higher parasitaemia threshold for pyrexia or a change in parasite dynamics that delays the parasites from reaching the pyrogenic threshold [39, 40].

The HbAS and HbAC target the pathogenic process of severe clinical malaria and influence non-severe clinical malaria risk [37]. The study observed a significantly lower IgG anti-PfEBA exposure in HbAS and HbAC compared with HbAA. Although, we do not fully understand different mechanisms in natural infections and how within-host parasite dynamics in the HbAA individuals and in AS or AC heterozygotes, the observation support previous finding that HbAC individuals frequently carry patent parasites, whereas HbAS individuals have reduced malaria positivity [36, 37]. Thus, lower exposure of *P. falciprum* parasite infections to the host immune system in HbAS results in low IgG antibody levels of EBA-175 compared to HbAC which sustains prolonged malaria infection with higher asexual parasite levels. Contrary to the finding of this study, some studies have reported enhanced IgG immune response specifically against *P. falciprum* erythrocyte membrane protein (PfEMP1) [41, 42]. A study conducted by Lell et al., demonstrated that the combination of naturally acquired immunity and sickle cell traits exhibited a remarkable synergistic effect, resulting in a substantial reduction in malaria symptoms [43].

## Conclusion

Symptomatic malaria patients with variant haemoglobin had significantly reduced IgG titres of gSG6-P1, Pfs230, and PfEBA-175 antibodies. The HbAS genotype is suggested to confer protection against malaria infection. Reduced exposure to infection ultimately reduces the induction of antibodies targeted against *P. falciparum* antigens.

## Data Availability

All the data are available in the manuscript.
